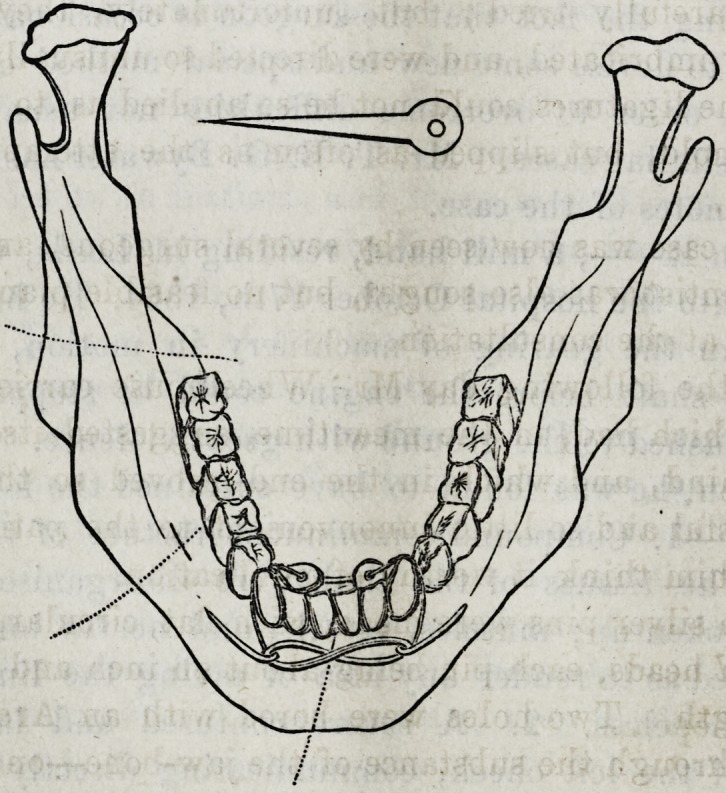# New Mode of Treating Complicated Fracture of the Lower Jaw—Simple and Compound Fracture of Lower Jaw; Compound Comminuted Fracture of Right Forearm; Amputation; Recovery

**Published:** 1868-02

**Authors:** 


### ARTICLE VI.

### *New Mode af treating Complicated Fracture of the Lower Jaw—Simple and Compound Fracture of Lower Jaw; Compound Comminuted Fracture of Right Forearm; Amputation; Recovery.*

The following case is worthy to be placed on record, not on account of the special features it presents, but also as illustrating the fact that the surgeon is occasionally called upon to devise some new and special method of treatment, in order to overcome difficulties not usually met with in similar cases. Mr. T. E. G. Bywater has obliged us with notes of the case.

Joseph R——, a mill hand, residing in Leeds, was admitted into the hospital October 27th, 1864. He had been caught in the gearing of machinery in motion, carried round a shaft before the engine could be stopped, and finally dashed to the ground with great violence. On examination, he was found to have sustained the following injuries:—1. Compound comminuted fracture of the forearm. The tissues of the limb were disorganised; the bones broken up; muscles, vessels, and nerves torn, and so crushed as to render any idea of saving the limb altogether hopeless. 2. A severe contused and lacerated wound of the left cheek, communicating directly with a fracture of the lower jaw, and extending upwards on the face for two inches and a half or three inches. The jaw had sustained three fractures: one at the symphysis, a second immediately in front of the insertion of the masseter muscle, and a third through the ramus of the bone and base of the coronoid process. The face and right side of the neck were much contused and ecchymosed. 3. There was a considerable amount of extravasated blood in and around the left orbit, and also between the sclerotic and conjunctiva of the eyeball of the same side; this effusion terminated abruptly at the margin of the cornea. There was no oozing of blood from either ear, nor from the nose, nor through the pharynx. The same evening, circular amputation of the forearm was performed about five inches below the point of the elbow, and the wound was brought together by wire sutures, and supported by strips of plaster and a bandage. The face was so swollen and bruised that no mechanical support could at this time be applied to the fractured jaw, and a little warm lead lotion was therefore ordered.

Oct. 30th.—Reaction has passed off and the patient is doing well; stump dressed: wound looks healthy, and is free from any appearance of sloughing.

The question of the treatment to be adopted in order to keep the fragments of the jaw in good position now came under consideration, and proved a very complicated one. The portion of the bone extending from the symphysis to the edge of the masseter was exceedingly loose, and, on examining it carefully, was observed to be so far dragged downwards by the action of the depressor muscles that the upper border of the incisor teeth of the fractured side was exactly on a level with the base of the bone on the sound one. It could be restored to its normal position without the application of any great amount of force, but extreme difficulty was experienced in retaining it there. The means that suggested themselves for this purpose were, first, the usual moulded external splint and bandage; but the use of any such appliance as this was rendered impossible by the presence of the large lacerated wound mentioned above. Secondly, lashing the teeth together by silver wire was very carefully tried; but, unfortunately, they were so closely imbricated, and were directed so unusually inwards that the ligatures could not be so applied as to maintain their hold, but slipped as often as the attempt was repeated.

The case was now seen by several surgeons, and the aid of a dentist was also sought, but no feasible plan was sugdested at the consultation.

On the following day Mr. Wheelhouse carried out an idea which had, in the meantime, suggested itself to his own mind, and which in the end proved so thoroughly successful and so little innonvenient to the patient as to make him think it worthy of publication.

Two silver pins were made, with flat, circular, and *perforated* heads, each pin being about an inch and a quarter in length. Two holes were bored with an Archimidean drill through the substance of the jaw-bone—one between the roots of the outer incisor and canine teeth of the unbroken side, and the second between the roots of the same teeth of the fractured side. Through these holes the two pins were passed *from behind forward,* the perforated heads, threaded with a good stout silk ligature, resting upon the floor of the mouth under cover of the frænum of the tongue. Having been well thrust forward through the drill-holes, the points were bent in opposite directions, the loose fragment was placed in good position, the ligature was brought forward over the teeth, and a figure-of-8 suture was then made round the reversed ends of the pins.

By this means perfect apposition was secured. The wound in the soft tissues of the face was allowed to granulate untrammeled by external pressure. The patient was enabled to take food easily and well, and, in short, made so excellent a recovery that he left the hospital on the 26th of November, exactly a month from the date of his admission, with his wounds all healed, his jaw moderately firm, and the line of his teeth perfect.—*London Lancet.*

---

## Figures and Tables

**Figure f1:**